# Femoral Aneurism

**Published:** 1829-05

**Authors:** 


### FEMORAL ANEURISM.

*Spontaneous Cure of Femoral Aneurism, aided by Pressure.* (WINCHESTER COUNTY HOSPITAL.)

JOHN LEAVY, ætat. forty-five, labourer, admitted with a large femoral aneurism of the left extremity; had been in the hospital two years previously under Mr. LYFORD, at which period he underwent the operation of having the right crural artery secured for a popliteal aneurism. The patient's account of his present affliction is as follows: In June last, while in the act of mowing, he felt something give way, or (as he expressed himself) snap in his thigh, which was productive of such excessive pain as to entirely prevent him proceeding with his employment. From this time a pulsation, or throbbing, commenced, which became so much aggravated at night as to deprive him of sleep. A very short time elapsed before a small tumor became distinct at the place where he felt the pulsation at the lower anterior and internal part of the thigh, which has gradually increased to its present enormous size, being four or five inches in circumference, circumscribed, rather hard, and can be almost entirely reduced by pressure on the artery above. The whole extremity much enfeebled; and he is quite incapacitated from moving without a stick or crutch. As he was desirous of making some domestic arrangements prior to his coming into the house, he became an out-patient. He was requested to make moderate pressure on the part by means of a flannel roller, and to keep himself at home perfectly quiet.

September 27.—He was made an in-patient. On examining the thigh, the tumor was found to have greatly subsided, and to have lost all pulsation; which, according to his statement, had taken place three days previously. He had experienced a most decided diminution of pain from the pressure of the bandage, which he therefore increased from time to time by tying a handkerchief very tight around the thigh, the knot of which was directly over the centre of the aneurism. Since the pulsations have ceased, he has felt exactly similar sensations to those with which he was troubled after the operation on the opposite limb. He now complains most severely of a burning heat immediately under the skin, which he compares to boiling water trickling down his foot and leg. May not this disordered feeling be connected or depend on the circulation of the parts below the aneurism being carried on by the more superficial vessels?

The pressure has been reapplied by means of a tourniquet and splint. The temperature of both feet exactly correspond.

October 8.—On removing the apparatus by which the pressure had been applied, all appearance of swelling had entirely vanished, and every vestige of disease removed. The knee-joint is now capable of the most perfect flexion and extension, and the patient enabled to walk without any support, and without inconvenience. He was therefore discharged cured.*

* Provincial Gazette, March.

